# Glycerol Improves Skin Lesion Development in the Imiquimod Mouse Model of Psoriasis: Experimental Confirmation of Anecdotal Reports from Patients with Psoriasis

**DOI:** 10.3390/ijms22168749

**Published:** 2021-08-15

**Authors:** Vivek Choudhary, Ismail Kaddour-Djebbar, Victoria E. Custer, Rawipan Uaratanawong, Xunsheng Chen, Elyssa Cohen, Rong Yang, Etsubdenk Ajebo, Sarah Hossack, Wendy B. Bollag

**Affiliations:** 1 Charlie Norwood VA Medical Center, Augusta, GA 30904, USA; vchoudhary@augusta.edu (V.C.); ismail.kaddour-djebbar@va.gov (I.K.-D.); 2 Department of Physiology, Medical College of Georgia at Augusta University, Augusta, GA 30912, USA; victoriaecuster@gmail.com (V.E.C.); rawipan_ua@yahoo.com (R.U.); schen@augusta.edu (X.C.); efcohen3@gmail.com (E.C.); yangrong_1223@hotmail.com (R.Y.); sarah.c.hossack@gmail.com (S.H.); 3 Department of Medicine (Dermatology), Faculty of Medicine Vajira Hospital, Navamindradhiraj University, Bangkok 10300, Thailand; 4 Department of Physiology, Medical School, Jianghan University, Wuhan 430050, China; 5 Department of Dermatology, Medical College of Georgia at Augusta University, Augusta, GA 30912, USA; eajebo@augusta.edu

**Keywords:** aquaporin-3 (AQP3), phospholipase D2 (PLD2), epidermis, glycerol, imiquimod (IMQ), keratinocytes, psoriasis, skin

## Abstract

Glycerol is used in many skin care products because it improves skin function. Anecdotal reports by patients on the National Psoriasis Foundation website also suggest that glycerol may be helpful for the treatment of psoriasis, although to date no experimental data confirm this idea. Glycerol entry into epidermal keratinocytes is facilitated by aquaglyceroporins like aquaporin-3 (AQP3), and its conversion to phosphatidylglycerol, a lipid messenger that promotes keratinocyte differentiation, requires the lipid-metabolizing enzyme phospholipase-D2 (PLD2). To evaluate whether glycerol inhibits inflammation and psoriasiform lesion development in the imiquimod (IMQ)-induced mouse model of psoriasis, glycerol’s effect on psoriasiform skin lesions was determined in IMQ-treated wild-type and PLD2 knockout mice, with glycerol provided either in drinking water or applied topically. Psoriasis area and severity index, ear thickness and ear biopsy weight, epidermal thickness, and inflammatory markers were quantified. Topical and oral glycerol ameliorated psoriasiform lesion development in wild-type mice. Topical glycerol appeared to act as an emollient to induce beneficial effects, since even in PLD2 knockout mice topical glycerol application improved skin lesions. In contrast, the beneficial effects of oral glycerol required PLD2, with no improvement in psoriasiform lesions observed in PLD2 knockout mice. Our findings suggest that the ability of oral glycerol to improve psoriasiform lesions requires its PLD2-mediated conversion to phosphatidylglycerol, consistent with our previous report that phosphatidylglycerol itself improves psoriasiform lesions in this model. Our data also support anecdotal evidence that glycerol can ameliorate psoriasis symptoms and therefore might be a useful therapy alone or in conjunction with other treatments.

## 1. Introduction

Psoriasis is a common skin ailment, with this chronic disease affecting approximately 2–3% of the worldwide population, including an estimated 7.5 million people in the United States ([1,2] and the National Psoriasis Foundation website). While not usually life threatening, psoriasis is associated with significant physical and psychological morbidity, with patients reporting a decrease in quality of life that is comparable to more serious chronic diseases [2,3]. Furthermore, individuals with the disease are predisposed to other co-morbidities [4,5,6], such as cardiovascular issues. Psoriasis is characterized by an activated immune system and inflammation [7], as well as hyperproliferation and abnormal differentiation of keratinocytes (reviewed in [8,9]). Activated keratinocytes produce immune cell-stimulating cytokines, and stimulated immune cells produce keratinocyte-activating agents. Accumulating evidence suggests that in certain susceptible individuals, immune cells and keratinocytes cross-talk to initiate and promote psoriasis (reviewed in [10,11,12]). Nevertheless, the exact cause of psoriasis is still unresolved, and this unclear understanding of its origin makes treatment a challenge. Indeed, individuals with psoriasis often express dissatisfaction with current therapies [13], due to lack of efficacy, side effects or expense. For example, biological agents used to treat psoriasis are costly and exhibit undesirable side effects, such as an increased risk of serious infections and possibly lymphoma [14]. 

Glycerol, also known as glycerin, is used in many lotions and ointments because it is known to improve dry skin and accelerate skin wound healing [15]. In fact, glycerin is advertised as a skin protectant that “helps prevent and protects chafed, chapped, cracked or windburned skin and lips.” Glycerol forms the backbone of several lipids, such as triglycerides and phospholipids, and when stored fat is used as a source of energy glycerol is released into the bloodstream. Glycerol entry into cells is facilitated by aquaglyceroporins such as aquaporin-3 (AQP3), which is expressed in skin and epidermal keratinocytes [16]. Glycerol has been thought to improve skin function through its ability to attract and retain water, as well as through its emollient properties; however, experiments with AQP3 knockout mice, as well as a sebum-deficient asebia mouse model, suggest that glycerol may have additional roles [17,18,19]. Indeed, anecdotal reports by psoriatic patients on the National Psoriasis Foundation website provide evidence that glycerol may possibly be useful for the treatment of psoriasis, although to date there are no experimental reports to confirm this idea. 

We have shown that AQP3 is physically and functionally associated with the lipid-metabolizing enzyme phospholipase-D2 (PLD2), which can convert the glycerol transported by AQP3 to the second messenger, phosphatidylglycerol [20,21,22]. We have further shown that phosphatidylglycerol can inhibit keratinocyte proliferation and stimulate keratinocyte differentiation [23]. Recently, we have shown that dioleoylphosphatidylglycerol, a specific phosphatidylglycerol species, decreases the expression of inflammatory mediators induced through the activation of toll-like receptor-2 and -4 (TLR-2 and TLR-4) in keratinocytes and a macrophage cell line [24]. Furthermore, dioleoylphosphatidylglycerol improves psoriasiform lesions in the imiquimod (IMQ)-induced mouse model of psoriasis [24]. Soy phosphatidylglycerol, a mixture of phosphatidylglycerol species, is also effective in reducing inflammation in a contact irritant ear edema mouse model [25], al-though our subsequent experiments examining its ability to inhibit TLR2- and TLR4-induced inflammatory mediator expression indicated that, in contrast to dioleoylphosphatidylglycerol, soy phosphatidylglycerol possesses a narrow therapeutic window, with higher doses actually stimulating the expression of some cytokines [26]. However, to our knowledge no systematic study has investigated glycerol’s effect on psoriasis. Here, we hypothesized that glycerol, as the precursor of phosphatidylglycerol, would inhibit keratinocyte proliferation and psoriasiform lesion development in the IMQ mouse model of psoriasis and that PLD2 would be required for this beneficial effect of glycerol.

## 2. Results

### 2.1. Topical Application of Glycerol Improved Psoriasiform Lesions, Reducing Inflammation and Epidermal Thickness in the IMQ Mouse Model of Psoriasis in Wild-Type Mice

**Figure 1:**
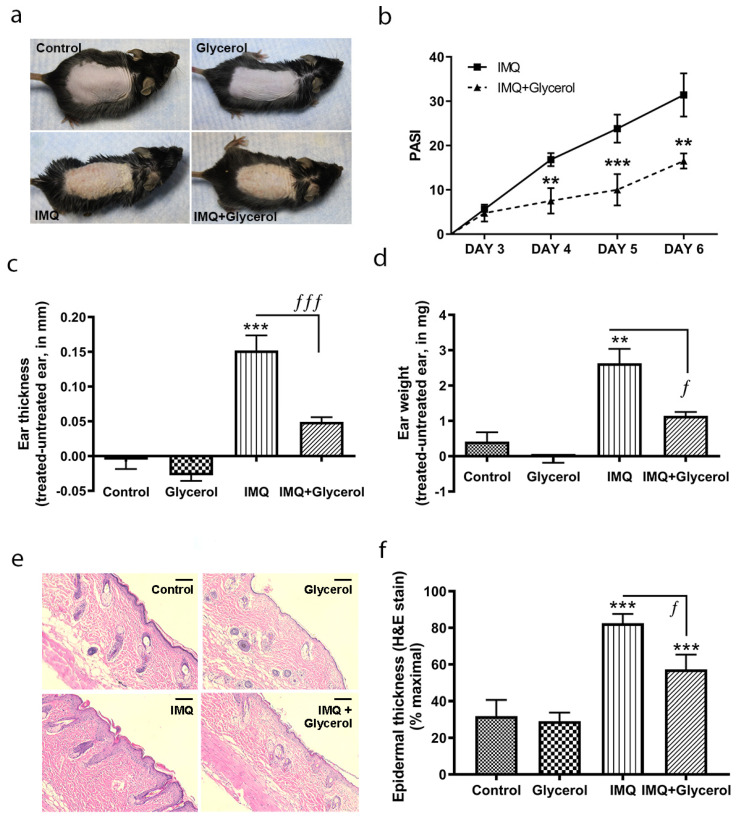
Topical glycerol improved IMQ-induced psoriasiform lesions in wild-type C57BL/6 mice. Daily for five days, five hours after IMQ or vehicle (petrolatum) application, water or glycerol was applied topically as a 50% (volume:volume) glycerol in water solution to wild-type C57BL/6 mice. (**a**) Representative photographs (day 6) showing effects on epidermal erythema, thickening and scaling with treatments. (**b**) Psoriasis area severity index (PASI) scores analyzed by unpaired, two-tailed t-tests comparing two groups within the same day. (**c**) Ear thickness and (**d**) ear biopsy weight measured as described in Methods. (**e**) Representative hematoxylin- and eosin (H&E)-stained micrographs showing back skin epidermal thickening with IMQ treatment (scale bar = 100 µm). (**f**) Quantitation of the thickness expressed as the percent maximal value, presented as means ± SEM (*n* = 4–5), with one-way analysis of variance (ANOVA) followed by Student–Newman–Keuls multiple-comparison post-hoc tests (GraphPad Prism, La Jolla, CA, USA) used to determine significant differences; ** *p* < 0.01, *** *p* < 0.001 versus control; ^ƒ^ *p* < 0.05, ^ƒƒƒ^ *p* < 0.001 as indicated.

The ability of glycerol to affect the development of psoriasiform lesions was investigated in the IMQ mouse model of psoriasis. This model was selected because IMQ is known to induce the development of psoriasis-like lesions in some human patients exposed to this agent for the treatment of skin conditions such as genital warts, actinic keratosis and non-operable superficial basal cell carcinoma [27]. Furthermore, anecdotal reports have suggested that topical glycerol application can improve psoriasis symptoms, and indeed, glycerol is a known emollient with beneficial effects to soften and soothe the skin. Therefore, we first tested the effects of glycerol administered topically. Every day for 5 days, 50% (volume:volume) glycerol in water or water alone was administered topically approximately 5 h after the topical application of IMQ or vehicle. Macroscopic observations indicated that IMQ-treated mice receiving glycerol topically exhibited less erythema, thickening and scaling than those treated with IMQ alone (Figure 1a), such that the IMQ-increased psoriasis area severity index (PASI) scores were significantly reduced with topical glycerol application (Figure 1b). Similarly, topical glycerol reduced IMQ-stimulated ear edema/inflammation, as measured by increases in ear thickness (Figure 1c), and inhibited the rise in ear weight (Figure 1d). Furthermore, topical application of glycerol also reduced epidermal thickness in harvested back skin in IMQ-treated animals compared to the IMQ-alone group (Figure 1e,f).

### 2.2. Topical Glycerol Improved IMQ-Induced Psoriasiform Lesions in PLD2 Knockout Mice

**Figure 2:**
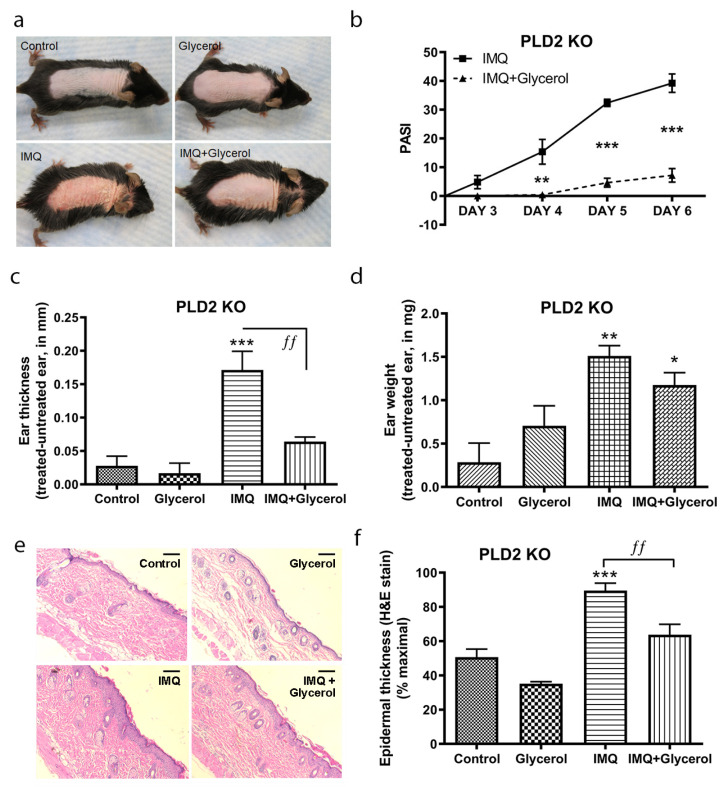
Topical glycerol improved IMQ-induced psoriasiform lesions in PLD2 knockout mice. As in Figure 1, IMQ or vehicle and water or glycerol (as a 50% glycerol in water solution) were applied topically to PLD2 knockout mice (on a C57BL/6 background). (**a**) Representative photographs (day 6) showing effects on epidermal erythema, thickening and scaling with treatments. (**b**) Psoriasis area severity index (PASI) scores analyzed by unpaired, two-tailed t-tests comparing two groups within the same day. (**c**) Ear thickness and (**d**) ear weight measured as described in Methods. (**e**) Representative H&E-stained micrographs showing back skin epidermal thickening with IMQ treatment (scale bar = 100 µm). (**f**) Quantitation of the thickness expressed as the percent maximal value, presented as means ± SEM (*n* = 4–5), with ANOVA followed by Student–Newman–Keuls multiple-comparison post-hoc tests used to determine significant differences; * *p* < 0.05, ** *p* < 0.01, *** *p* < 0.001 versus control; ^ƒƒ^ *p* < 0.01 as indicated; *n* = 4–5.

Glycerol transported into the cells by AQP3 can be converted to phosphatidylglycerol by PLD2 [19], and we have previously observed an ability of phosphatidylglycerol to ameliorate psoriasiform skin lesions in the IMQ-induced model, presumably through its ability to inhibit innate immune system recruitment and inflammation by blocking toll-like receptor activation [24]. Therefore, it was not clear whether the beneficial effect of topical glycerol in wild-type mice was due to its emollient properties or to its PLD2-mediated conversion to the second messenger phosphatidylglycerol. In order to investigate the involvement of PLD2 and its enzymatic conversion of glycerol to phosphatidylglycerol in the effect of topical glycerol, we therefore repeated our experiments in PLD2 knockout mice where glycerol’s conversion to phosphatidylglycerol would be inhibited. As expected, PLD2 mRNA expression was essentially absent in the back skin of PLD2 knockout mice compared to the control mice (Figure S1). Macroscopically, the IMQ-induced skin lesions were improved in the PLD2 knockout mice that were topically treated with glycerol and IMQ compared to the IMQ-alone group in terms of erythema, thickening and scaling (Figure 2a). PASI scores were also significantly reduced in the IMQ plus topically-applied glycerol group as compared to IMQ alone (Figure 2b). Similarly, topical glycerol significantly reduced the IMQ-induced increase in ear thickness indicative of ear edema/inflammation (Figure 2c) as well as the epidermal thickness of the back skin (Figure 2e,f). Topical glycerol application also appeared to decrease IMQ-induced ear weight compared to the IMQ alone group, but this effect did not achieve statistical significance (Figure 2d). These results in PLD2 knockout mice suggest that PLD2 is not required for topical glycerol’s action, as topical glycerol improves psoriasiform lesions even in PLD2 knockout mice. However, since AQP3-mediated transport of hydrogen peroxide is known to promote a psoriasiform phenotype [28], a possible mechanism by which topical glycerol might inhibit psoriasiform lesion development is through competition with hydrogen peroxide for transport through AQP3. Indeed, we found that intracellular ROS levels in primary cultures of mouse keratinocytes exposed to hydrogen peroxide were significantly lower in the presence of glycerol (Figure S2). This suggests that topically applied glycerol, in addition to its action as an emollient, might also competitively inhibit hydrogen peroxide transport through AQP3 to improve the psoriasiform lesions induced by IMQ. Alternatively, it is possible that topical application of glycerol might have removed/diluted the IMQ applied 5 h earlier, although why such removal/dilution would only occur with glycerol/water application and not with water alone in control IMQ-treated mice is not clear.

### 2.3. Oral Glycerol Improved IMQ-Induced Psoriasiform Lesions in Wild-Type C57BL/6 Mice

**Figure 3:**
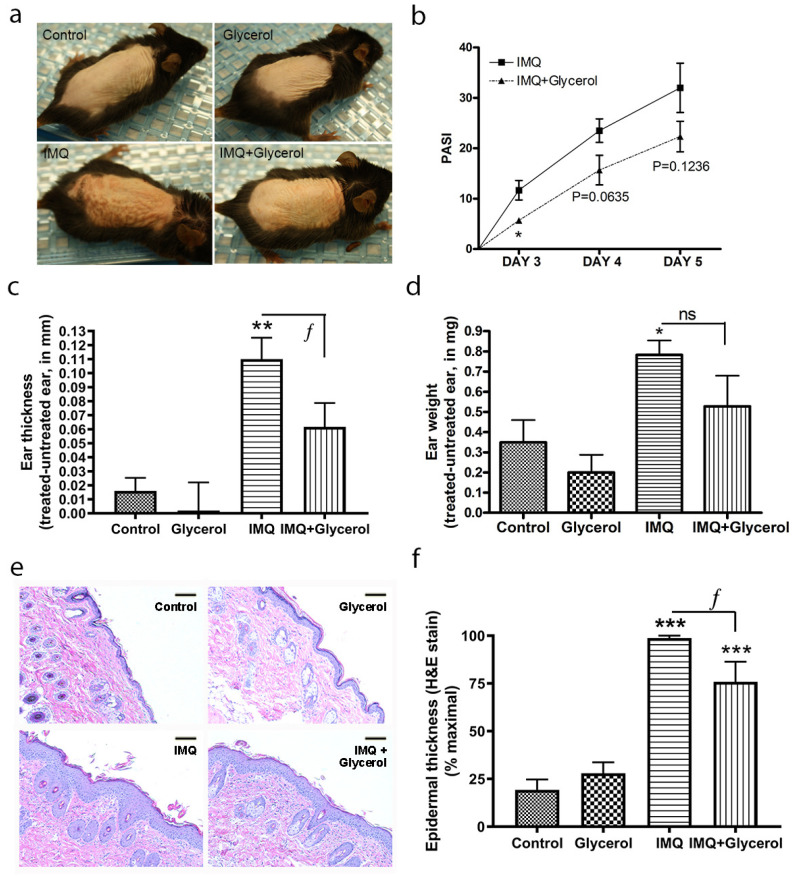
Oral glycerol improved IMQ-induced psoriasiform lesions in wild-type C57BL/6 mice. Mice receiving glycerol (2%) in drinking water or not were treated with vehicle or IMQ daily for 5 days. (**a**) Representative photographs (day 6) showing effects on epidermal erythema and scaling with treatments. (**b**) Psoriasis area severity index (PASI) scores analyzed by unpaired, two-tailed t-test comparing two groups within the same day. (**c**) Ear thickness and (**d**) ear biopsy weight measured as described in Methods. (**e**) Representative H&E-stained micrographs showing back skin epidermal thickening with IMQ treatment (scale bar = 100 µm). (**f**) Quantitation of the thickness expressed as the percent maximal value, presented as means ± SEM (*n* = 4–5). ANOVA with Student–Newman–Keuls multiple-comparison post-hoc tests was used to determine significant differences; * *p* < 0.05, ** *p* < 0.01, *** *p* < 0.001 versus control; ^ƒ^ *p* < 0.05 as indicated.

Glycerol should not act as an emollient (and/or remove/dilute IMQ) when administered orally; therefore, we decided to administer glycerol orally instead to determine its effect on IMQ-induced psoriasiform lesions. In addition, oral glycerol should be transported to the epidermis via physiological mechanisms as opposed to the pharmacological application of high concentrations of topical glycerol. Indeed, it is known that providing glycerol in drinking water can reverse the skin phenotypic changes observed in AQP3 knockout mice [29], indicating that orally administered glycerol arrives at the correct site of action to affect epidermal keratinocytes. Mice were administered 2% glycerol in their drinking water beginning on the day of shaving (two days before the first imiquimod/vehicle application). Mice receiving this oral glycerol in addition to IMQ treatment showed less erythema, thickening and scaling than those treated with IMQ alone (Figure 3a), and PASI scores, estimated by an experienced dermatologist in a blinded manner, confirmed the idea that glycerol ameliorates psoriasiform lesion development at earlier times of model generation (Figure 3b). Similarly, oral glycerol reduced IMQ-stimulated ear edema/inflammation, as measured by ear thickness, in response to IMQ (Figure 3c), and inhibited the increase in ear weight, returning a significantly enhanced ear weight with IMQ alone to a value that was not significantly different from the control in the presence of oral glycerol (Figure 3d). Epidermal thickness was measured in harvested back skin; as shown in Figure 3e and quantified in Figure 3f, oral glycerol significantly inhibited the increase in epidermal thickness induced by IMQ treatment. For all parameters measured, glycerol alone had no significant effect, including on body weight (Figure S3). 

### 2.4. Oral Glycerol Did Not Improve Psoriasiform Skin Lesions in PLD2 Knockout Mice

**Figure 4:**
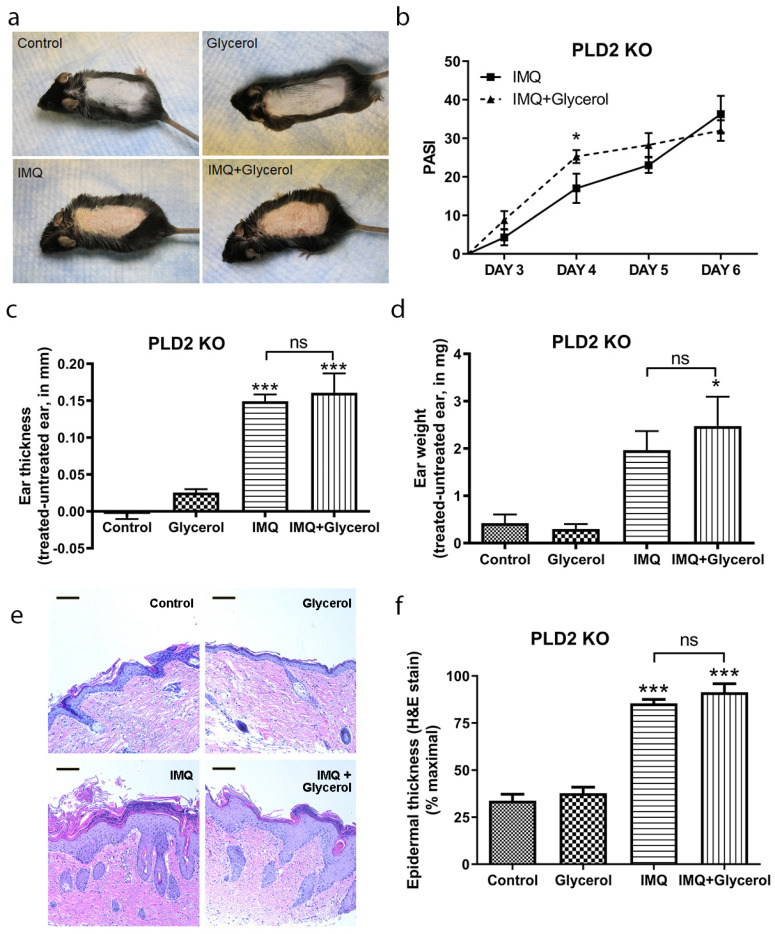
Oral glycerol does not improve IMQ-induced psoriasiform lesions in PLD2 knockout mice. PLD2 knockout mice receiving glycerol (2%) in drinking water or not were treated with vehicle or IMQ daily for 5 days. (**a**) Representative photographs (day 6) showing effects on epidermal erythema and scaling with treatments. (**b**) Psoriasis area severity index (PASI) scores were quantified and analyzed by unpaired, two-tailed t-tests comparing two groups within the same day. (**c**) Ear thickness and (**d**) ear weight measured as described in Methods. (**e**) Representative H&E-stained micrographs showing back skin epidermal thickening with IMQ treatment (scale bar = 100 µm). (**f**) Quantitation of the thickness expressed as the percent maximal value, presented as means ± SEM (*n* = 4–5). ANOVA with Student–Newman–Keuls multiple-comparison post-hoc tests was used to determine significant differences; * *p* < 0.05, ** *p* < 0.01, *** *p* < 0.001 versus control; *n* = 4–5; ns = non-significant.

We also supplied glycerol orally and performed IMQ experiments in PLD2 knockout mice, where the PLD2-mediated conversion of glycerol to phosphatidylglycerol should be prevented. Unlike with oral glycerol in wild-type mice, macroscopic skin lesions were not improved by oral glycerol administration in PLD2 knockout mice (Figure 4a), and, if anything, the PASI scores were higher (not lower) initially in the oral glycerol-treated IMQ group compared to IMQ alone mice (Figure 4b), although at the end of the experiment PASI scores were comparable in both of the IMQ-treated groups. The IMQ-induced increase in ear thickness and ear weight was not different in PLD2 knockout mice treated with or without oral glycerol (Figure 4c,d). Similarly, the IMQ-induced increase in the epidermal thickness of the back skin was not inhibited by oral glycerol in PLD2 knockout mice (Figure 4e,f). These results suggest that PLD2 is essential for glycerol’s action when administered orally, presumably because glycerol conversion to phosphatidylglycerol is necessary for glycerol’s action on psoriasiform lesions in the absence of its emollient (or IMQ removal/dilution or pharmacologic topical) effect. 

### 2.5. Oral Glycerol Inhibits the IMQ-Induced Increase in Molecular Markers of Psoriasis in Wild-Type C57BL/6 Mice

**Figure 5:**
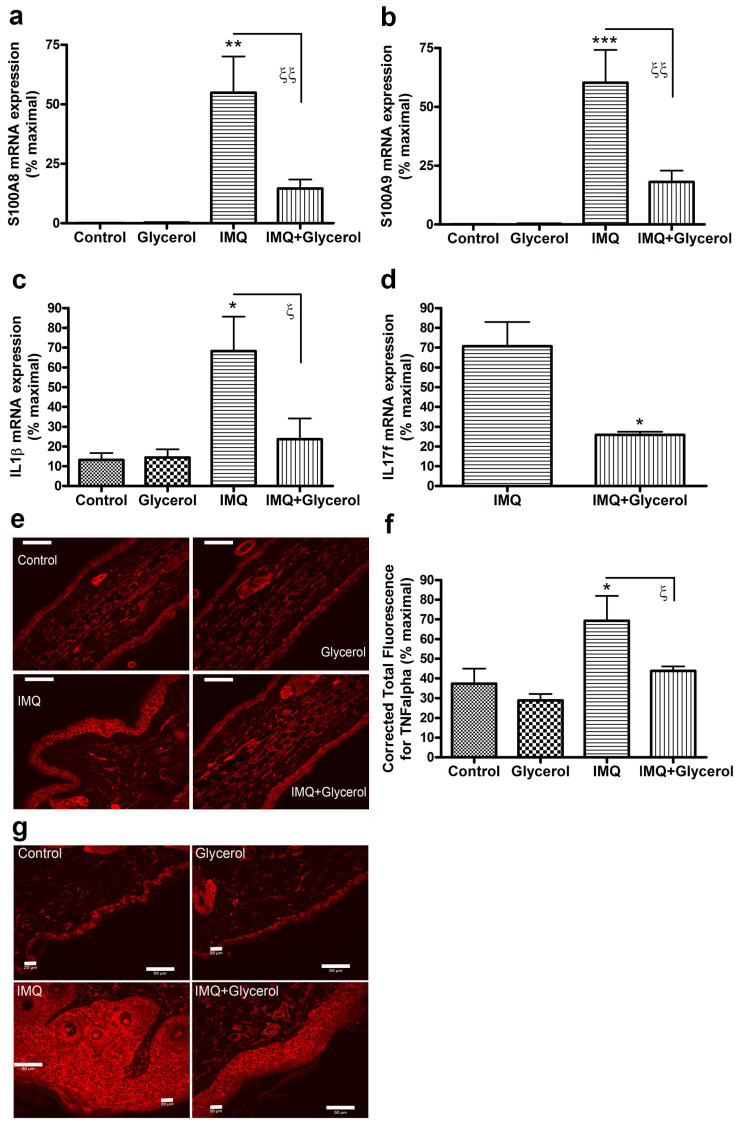
Oral glycerol reduces the IMQ-induced expression of anti-microbial peptides and inflammatory mediators in wild-type C57BL/6 mice. Mouse skin was homogenized and RNA isolated as described in Methods. Quantitative RT-PCR analysis for (**a**) S100A8, (**b**) S100A9, (**c**) IL-1β, and (**d**) IL-17f was performed using the delta-delta Ct method with Gapdh and/or Rplp0 as the endogenous control; results represent the means ± SEM expressed as percent maximal. (Please note that the mRNA levels of IL-17f were undetectable in the absence of IMQ treatment.) (**e**) Representative micrographs of formalin-fixed, paraffin-embedded ear skin from mice treated as indicated and stained for TNFα (scale bars as indicated); (**f**) quantitation of TNFα immunostaining. (**g**) Formalin-fixed, paraffin-embedded back skin from mice treated as indicated stained for TNFα. ANOVA with Student–Newman–Keuls multiple-comparison post-hoc tests was used to determine significant differences. For IL-17 data analysis, a two tailed t-test was used. * *p* < 0.05, ** *p* < 0.01, *** *p* < 0.001 versus control; ^ξ^ *p* < 0.05, ^ξξ^ *p* < 0.01 as indicated; *n* = 4–5.

Based on the results obtained with oral glycerol in wild-type mice versus PLD2 knockout mice, we sought to further characterize the effects of oral glycerol on other markers of psoriasis in wild-type mice. S100A proteins represent members of a family of antimicrobial peptides that are upregulated in psoriatic skin and in animal models of psoriasis [30,31]. Indeed, S100A protein levels are known to improve with effective treatment [32,33,34]; similarly, cytokines that are elevated in psoriasis also induce the expression of S100 proteins [9,30,34]. As expected based on the literature, IMQ induced the expression of S100A8 and S100A9 in skin; more importantly, however, glycerol significantly inhibited this increase (Figure 5a,b). Interleukin-1beta (IL-1β) is a pro-inflammatory cytokine also known to be upregulated and to play a role in psoriasis [8], as well as to regulate immune function [35]. As with the S100A proteins, IMQ increased mRNA levels of IL-1β, and glycerol reduced this effect (Figure 5c). The levels of the cytokine IL-17 are also elevated in psoriasis [36,37] and an anti-IL-17 medication (Ixekizumab; brand name Taltz) recently approved by the Food and Drug Administration has shown efficacy for the treatment of psoriasis [38]. We found that IMQ induced IL-17f expression and glycerol significantly inhibited this induction (Figure 5d). IMQ also increased IL-17a expression but levels of this transcript were quite low (a cycle threshold of approximately 35) and were unaltered with glycerol treatment (data not shown). Finally, tumor necrosis factor-alpha (TNFα) is a key cytokine that is upregulated in psoriasis; indeed, anti-TNFα medications have been successfully used to treat psoriasis [12]. TNFα protein levels were elevated in IMQ-treated ear epidermis, and glycerol significantly inhibited this increase (Figure 5e,f). TNFα protein level was also increased with IMQ treatment in back skin and this increase was inhibited by glycerol as well (Figure 5g). Together, these results suggest that oral glycerol can also be beneficial in improving IMQ-induced psoriasiform lesions and suggest that it acts by conversion to phosphatidylglycerol, which has previously been shown to have anti-inflammatory actions in keratinocytes in vitro and skin in vivo.

## 3. Discussion

Our results provide experimental evidence that glycerol improves psoriasiform lesions and inhibits pro-inflammatory cytokine expression in the IMQ mouse model of psoriasis. This model was selected because IMQ is known to induce the development of psoriasis in certain human patients exposed to this agent [27], suggesting parallels with psoriasis. Indeed, IMQ-treated C57BL/6 mice show changes in gene expression similar to those observed in psoriasis [39,40]. Our data support patients’ anecdotal evidence that glycerol is able to ameliorate psoriasis symptoms either alone or in conjunction with other agents and provide two potential mechanisms, in addition to its emollient properties, for glycerol’s action: (a) inhibition of hydrogen peroxide uptake into cells and (b) conversion to anti-inflammatory phosphatidylglycerol. This result is consistent with previous results in which topical glycerol (and/or xylitol) inhibits irritation and inflammation induced by sodium lauryl sulfate [41,42], as well as data indicating that a lotion supplemented with glycerol is more efficacious in atopic dermatitis than an unsupplemented lotion [43]. Moreover, glycerol is widely added to many skin lotions as a moisturizer and is already used to treat or prevent dry, rough, scaly, itchy skin and minor skin irritations like diaper rash, as well as skin burns from radiation therapy [44,45]. In an in vivo study, application of glycerol was reported to increase transmission of ultraviolet light through psoriatic plaques by roughly 2-fold by decreasing its backscatter [46], suggesting another potential beneficial effect of glycerol in treating psoriasis, since ultraviolet radiation can be used therapeutically to improve psoriasis [47].

Our results also suggest a beneficial effect of topically applied glycerol on psoriasiform lesions. However, this favorable effect of topical glycerol was observed even in the absence of PLD2 (in PLD2 knockout mice), suggesting that it could be due to glycerol’s emollient effect or some other mechanism. For example, glycerol may improve barrier function of the epidermis, as reported previously [42,48]; since barrier integrity is known to regulate inflammation in the skin [49,50], this effect could result in amelioration of skin lesions. Nevertheless, the mechanism by which glycerol promotes barrier integrity is unknown, although in possibly related results, our laboratory has previously shown that transgenic mice over-expressing AQP3 under control of the human keratin 1 promoter exhibit accelerated barrier repair after disruption [19]. 

The beneficial effect of topical glycerol could also be due to competitive inhibition of hydrogen peroxide transport through AQP3, as we have shown that glycerol application decreased hydrogen peroxide-induced intracellular ROS levels in primary cultures of mouse keratinocytes (Supplemental Figure S1). This result suggests that AQP3′s transport of hydrogen peroxide in certain skin conditions might also be reversed by application of glycerol. Hara-Chikuma et al. [28] have shown that AQP3-mediated transport of hydrogen peroxide promotes a psoriasiform phenotype in two mouse models of psoriasis, an IL-23-induced ear edema model and the IMQ model. Thus, these authors showed a reduction in ear inflammation in response to IL-23 injection in AQP3 knockout mice, and in supplementary data, an inhibition of IMQ-induced epidermal thickening [28]. In these studies it was shown, using wild-type and AQP3 knockout bone marrow chimeric mice, that AQP3 ablation in keratinocytes, rather than hematopoietic cells, was associated with reduced inflammation due to inhibited influx of hydrogen peroxide into the epidermal cells [28]. Further studies are clearly needed to examine these possibilities; nevertheless, our data support anecdotal evidence that glycerol can ameliorate psoriasis symptoms.

We recently showed that topical application of dioleoylphosphatidylglycerol improved IMQ-induced psoriasiform skin lesions in mice [24], suggesting that the beneficial effect of glycerol could be due to its conversion to phosphatidylglycerol. It is thought that phosphatidylglycerol reduces inflammation and improves psoriasiform skin lesions by decreasing inflammatory mediator production induced by TLR2 and TLR4 activation in response to danger-associated molecular patterns, such as anti-microbial peptides [24], many of which are elevated in psoriasis and mouse models of the disease [31,51]. We, therefore, used glycerol orally to avoid its emollient and topical pharmacologic effect and to investigate if its conversion to phosphatidylglycerol is one of the possible molecular mechanisms involved in its amelioration of psoriasiform lesions. Indeed, when we administered glycerol orally in PLD2 knockout mice we did not see any improvement in the IMQ-induced psoriasiform lesions. Thus, intact PLD2 in wild-type mice was required for orally administered glycerol to impart any beneficial effect. Phospholipase D2 (PLD2) is a lipolytic enzyme implicated in a variety of cellular processes, including cell proliferation and differentiation [19,22]. Previously, we showed that AQP3 and PLD2 co-localize in caveolin-rich membrane microdomains in keratinocytes to allow the production of phosphatidylglycerol [21]. Since oral glycerol was beneficial in wild-type but not in PLD2 knockout mice, our results also suggest that, in addition to its emollient effect and possible inhibition of hydrogen peroxide influx, glycerol benefits psoriasiform lesions by its PLD2-mediated conversion to phosphatidylglycerol. On the other hand, in PLD2 knockout mice, oral glycerol actually worsened psoriasiform lesions, at least early on in lesion development. We are unsure of the reason for this apparent transient exacerbation, although we speculate that it might be the result of glycerol’s use as a substrate to produce ATP, thereby enhancing keratinocyte proliferation [52]. 

Our results demonstrate that glycerol can also inhibit pro-inflammatory cytokine mRNA and protein expression in the IMQ model (Figure 5). Similar to our data, there is emerging evidence of anti-inflammatory actions of glycerol from other laboratories [42,53]. In one such report, the HaCaT keratinocyte cell line was exposed to gram-positive *Streptococus mutans* for 24 h and challenged with probiotic *Lactobacillus reuteri* supplemented with or without glycerol. Glycerol supplementation enhanced *Lactobacillus reuteri*-induced suppression of pro-inflammatory IL-8 and human beta-defensin-2 expression, suggesting a possible anti-inflammatory action of glycerol [53]. Similarly, treatment of SKH1 hairless mouse skin with 10% glycerol inhibited sodium lauryl sulfate-induced expression of IL-1β and TNFα [42]. Since our results suggest the involvement of PLD2 in glycerol’s anti-inflammatory actions, it seems possible that PLD2 may also play a role and contribute to the effects seen in these other studies.

In conclusion, these results provide experimental evidence for the idea that glycerol might be useful in treating psoriasis. Topical administration of glycerol seems the most likely avenue for its use to treat psoriasis, because it can then act as an emollient, a hydrogen peroxide transport inhibitor and a precursor for the formation of phosphatidylglycerol, although in this study, oral glycerol was used as a tool to determine whether its PLD2-mediated conversion to phosphatidylglycerol was responsible for glycerol’s beneficial effects. Since other medications, such as anti-TNFα agents, have possible serious side effects, which include reactivation of latent tuberculosis, increased risk of serious infections and lymphoma, hepatotoxicity and worsening of congestive heart failure [54], our results suggest that glycerol may be useful for the long-term treatment of psoriasis, perhaps in mild psoriasis or after the disease is brought under control using biologicals or other therapies. On the other hand, single nucleotide polymorphisms in PLD2 have been associated with several types of cancer and hypertension [55,56], suggesting that there are likely individual variations in the activity of this enzyme. In this case glycerol might be ineffective in individuals with lower PLD2 activity, since PLD2 activity is required to convert the glycerol transported by AQP3 to phosphatidylglycerol. In contrast, administering phosphatidylglycerol itself would bypass the requirement for PLD2 and might be more universally effective. Our data suggest that future studies on the topical treatment of psoriasis patients with glycerol and/or phosphatidylglycerol are warranted, ideally in a randomized, double-blind, placebo-controlled fashion.

## 4. Methods

### 4.1. Animal Experiments

All animal protocols were approved by the Augusta University (protocol #15-0725) or Charlie Norwood VA Medical Center (protocol # 19-04-113) Institutional Animal Care and Use Committees and were conducted according to National Institutes of Health guidelines for the Care and Use of Laboratory Animals. The IMQ-induced psoriasis model was generated as in [24] in approximately 10-week-old wild-type (WT) C57BL/6 mice or PLD2 knockout (KO) mice on a C57BL/6 background, generously provided by Dr. Michael Frohman (Stony Brook University, Stony Brook, NY, USA), who obtained them from their creator, Dr. Gil Di Paolo (Columbia University, New York, NY, USA). Briefly, the backs of male WT or PLD2 KO mice, between the ages of 8 and 10 weeks, were shaved and depilated under isoflurane anesthesia. For the oral glycerol experiments, mice were at this time provided with plain water or water containing 2% glycerol. Two days later psoriasiform lesion development was initiated by topical application of 62.5 mg of Aldara cream (Meda Pharmaceuticals, Somerset, NJ, USA) or a vehicle (Vaseline) to the back and right ear of the mice. Vehicle or Aldara were applied daily for an additional four days, with daily skin lesion monitoring. On the sixth day mice were sacrificed and ear edema/inflammation was monitored using a digital caliper to measure thickness and an AX26 DeltaRange microbalance (Mettler Toledo, Columbus, OH, USA) to determine the weight of a 4 mm punch biopsy of the ears, which was then formalin-fixed. A portion of back skin was also fixed in formalin for paraffin embedding, sectioning and staining with H&E to monitor epidermal thickness and in some cases, TNFα immunoreactivity. From a subset of animals (3 mice per each control group and 5 mice per each imiquimod-treated group), back skin was also harvested and flash-frozen for subsequent homogenization, RNA isolation and quantitative RT-PCR analysis of gene expression (see below).

### 4.2. Histology and Measurement of Epidermal Thickness

Sections (5 µm) were cut from formalin-fixed, paraffin-embedded skin blocks. Slides were processed and stained with H&E via standard histological procedures. Photomicrographs of the epidermis of each section were taken randomly and analyzed to determine epidermal thickness using ImageJ (National Institutes of Health, Bethesda, MD, USA) by three observers who were blinded to the sample identity, as described previously [24]. 

### 4.3. TNFa Immunoreactivity

Sections prepared from formalin-fixed paraffin-embedded ear biopsies or back skin as above were deparaffinized and rehydrated as described previously [57]. Sections were processed for antigen retrieval, inhibition of endogenous peroxidase with hydrogen peroxide, and blocking of non-specific antibody binding. They were then incubated with anti-TNFα antibody (Novus Biologicals, Littleton, CO, USA), and immunoreactivity was visualized with a Cy5-conjugated secondary antibody. Staining was performed by Georgia Pathology Research Services (Augusta, GA, USA) using standard protocols. TNFα staining was determined in multiple random sections using ImageJ analysis and quantified in terms of fluorescent intensity in the demarcated area of the epidermis.

### 4.4. RNA Isolation

Using a mortar and pestle frozen skin tissue was homogenized under liquid nitrogen. The resulting powder was solubilized and RNA was isolated using TRIzol (ThermoFisher Scientific, Waltham, MA, USA) as per the manufacturer’s protocol. The total RNA was checked for quality and quantified using a Nanodrop instrument (ThermoFisher Scientific). One microgram of total RNA was used to generate complementary DNA using ABI High-Capacity cDNA Reverse Transcription kits (ThermoFisher Scientific). Quantitative PCR was then performed in a StepOnePlus (ThermoFisher Scientific) instrument using Taqman primer-probe sets and Taqman reagents (ThermoFisher Scientific) as per the supplier’s instructions. The primer-probe sets used were: S100a9 (Mm00656925_m1), Il1b (Mm00434228_m1), Il6 (Mm00446190_m1), Il17a (Mm00439618_m1), Il17f (Mm00521423_m1) and Tnfα (Mm00443258_m1), with the mouse Gapdh (Mm99999915_g1) and Rplp0 (Mm00725448_s1) genes used as endogenous housekeeping genes for delta-delta Ct analysis.

### 4.5. Statistical Analysis

Differences were determined using one-way analysis of variance (ANOVA) followed by Newman–Keuls post-hoc tests on the number of mice (i.e., the n) indicated in each figure legend; statistical significance was assigned at *p* < 0.05. Statistical analyses were performed using GraphPad Prism (San Diego, CA, USA). 

## Data Availability

The data presented in this study are available in the article and supplementary materials.

## Supplementary Materials

The following are available online at https://www.mdpi.com/article/10.3390/ijms22168749/s1.
