# Supplementary material for: Glycerol Improves Skin Lesion Development in the Imiquimod Mouse Model of Psoriasis: Experimental Confirmation of Anecdotal Reports from Patients with Psoriasis
Source: Int J Mol Sci. 2021 Aug 15;22(16):8749. doi: 10.3390/ijms22168749 (PMC8395744; doi:10.3390/ijms22168749)
Supplement: Supplementary file 1 [file ijms-22-08749-s001.zip › ijms-1340741-supplementary.pdf]

# SUPPLEMENTARY MATERIAL

Glycerol improves skin lesion development in the imiquimod mouse model of psoriasis:

Experimental confirmation of anecdotal reports from patients with psoriasis

Vivek Choudhary, Ismail Kaddour-Djebbar, Victoria E. Custer, Rawipan Uaratanawong, Xunsheng Chen, Elyssa Cohen, Rong Yang, Etsubdenk Ajebo, Sarah Hossack and Wendy B. Bollag

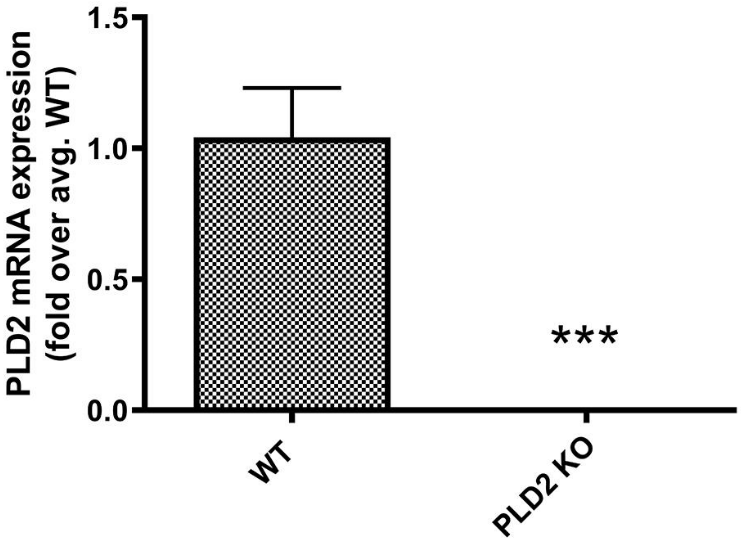


**Figure S1: PLD2 mRNA expression in back skin of the PLD2 knockout mice.** Mouse back skin from WT and PLD2 knockout mice was homogenized as described in Methods and RNA isolated. Quantitative RT-PCR analysis for PLD2 was performed using the delta-delta Ct method with Gapdh and Rplp0 as the endogenous control. Results represent the means ± SEM expressed as percent maximal (n= 4-6). Two tailed t-test was used to determine statistically significant differences, ***p<0.001 versus WT.

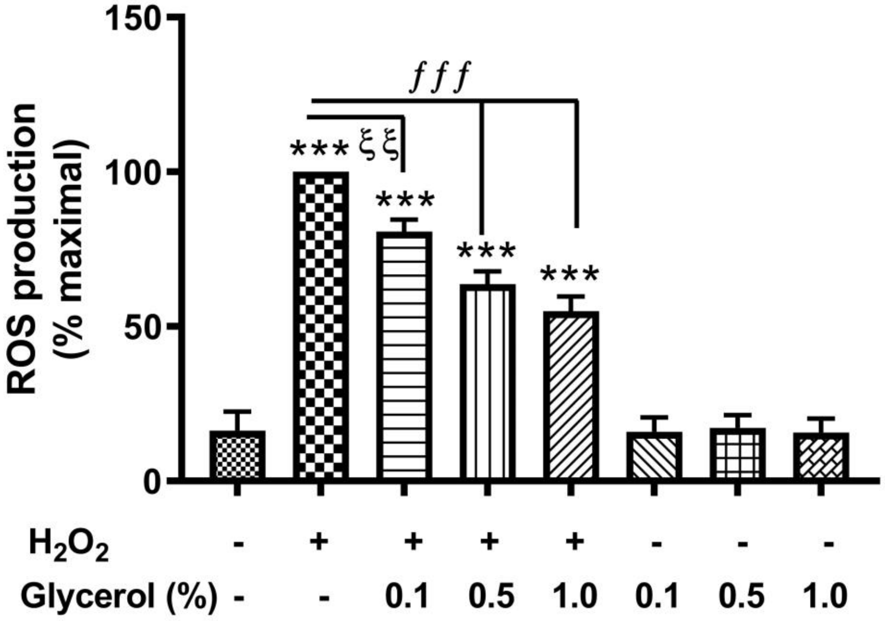


**Figure S2: Glycerol inhibits hydrogen peroxide transport, as measured by intracellular ROS levels, in primary cultures of mouse keratinocytes.** To measure hydrogen peroxide entry into cells, OxiSelect Intracellular ROS Assay kit (#STA-342, Cell Biolabs, Inc., San Diego, CA) was used as per the manufacturer's protocol. Briefly, primary cultures of mouse keratinocytes were plated on black cell culture fluorometric 96-well plates. The next day cells were washed twice with DPBS and 100 μL of 1x DCFH-DA/media solutions were added and incubated for 30 minutes. Cells were washed twice with DPBS and then treated with 100 μM hydrogen peroxide ($H_2O_2$) with or without glycerol (0.1 or 0.5 or 1.0%) as indicated for 1 hour. Fluorescence (intracellular ROS levels) was read by a fluorometric plate reader at 480 nm/530 nm from 5 min to 60 mins every 5 minutes and averaged from all time points. The ROS levels (fluorescence) are shown here as percent maximal and represent means ± SEM (n=3). One-way analysis of variance with Student-Newman-Keuls multiple comparison post-hoc tests (GraphPad Prism, La Jolla, CA) was used to determine significant differences; ***p<0.001 versus control; $^{\xi\xi}$p<0.01 or $^{fff}$p<0.001 as indicated.

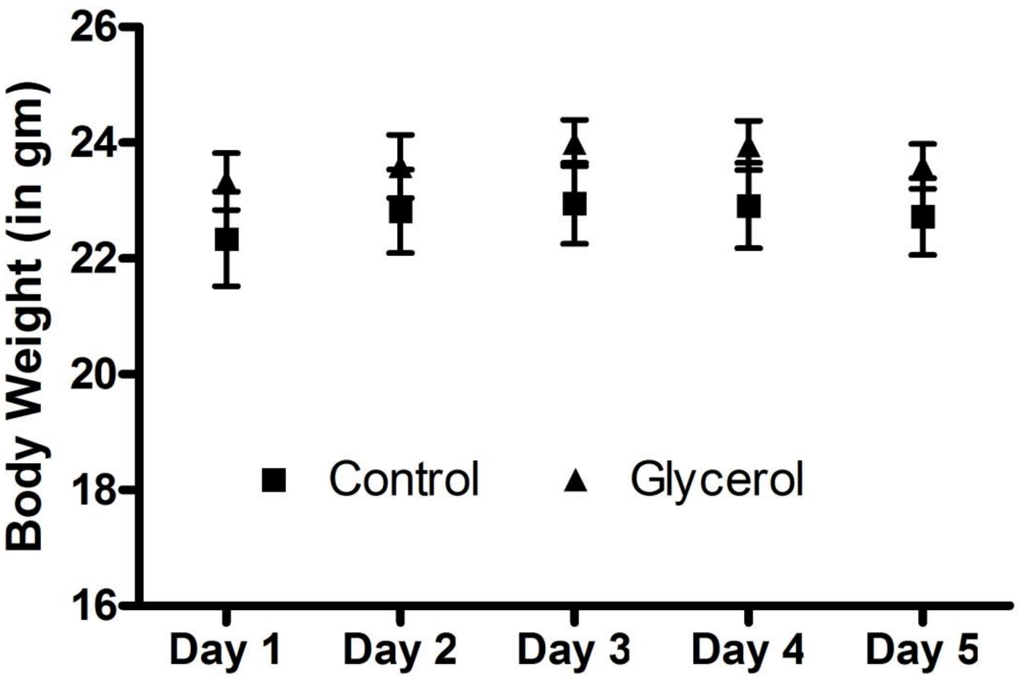


**Figure S3: Oral glycerol does not affect mouse body weight.** C57BL/6 mice receiving glycerol in the drinking water or not were treated with vehicle (petrolatum) or IMQ daily for 5 days. Body weights (in grams) of mice were recorded every day for 5 days. The quantitative data are presented as means ± SEM (n=4-5). One-way analysis of variance with Student-Newman-Keuls multiple comparison post-hoc tests (GraphPad Prism, La Jolla, CA) was used to determine significant differences. The body weights of glycerol-fed mice were not different from control mice on any day.
